# Kipiyecipakiciipe (“coming home”): a study protocol for a multi-method investigation of culturally grounded resilience against substance-use among Shawnee adults

**DOI:** 10.3389/fpubh.2025.1663919

**Published:** 2025-10-30

**Authors:** Evan J. White, Ryan Tomm, Victoria M. O'Keefe, Danielle L. Bethel, Gabe Cochran, Makiah Torres, Fiona Grubin, Maisie Conrad, Nicole R. Baughman, Andrea Wiglesworth, Xi Ren, Wesley Vaught

**Affiliations:** ^1^Laureate Institute for Brain Research, Tulsa, OK, United States; ^2^Oxley School of Community Medicine, University of Tulsa, Tulsa, OK, United States; ^3^Center for Indigenous Health, Department of International Health, Johns Hopkins Bloomberg School of Public Health, Baltimore, MD, United States; ^4^Department of Psychology, Oklahoma State University, Tulsa, OK, United States

**Keywords:** substance-use disorders, indigenous-health, neuroimaging, community readiness, qualitative methods, mixed-methods, tribal sovereignty, population health

## Abstract

**Background:**

American Indian and Alaska Native (AI/AN) peoples bear the highest U.S. burden of substance-use disorder (SUD) and drug-overdose mortality. Mechanistic evidence linking community-defined cultural protective factors to neurobehavioral pathways of SUD risk is virtually absent. Most AI/AN health studies are descriptive and rarely integrate neuroscience.

**Objectives:**

The Kipiyecipakiciipe (“coming home”) project aims to (i) operationalize Shawnee-defined cultural engagement variables, (ii) test their associations with neurobehavioral markers of reward, decision-making, and self-regulation, and (iii) establish evidence base for tribally guided prevention and recovery strategies.

**Methods:**

Guided by community-based participatory research, the three-phase multi-method protocol combines qualitative inquiry, a Community Readiness Assessment (CRA), and multimodal neuroimaging. *Phase 1* included 22 Shawnee adults and employed *N* = 3 focus-group discussions (including Nominal Group Technique) and *N* = 1 in-depth interview to generate an operational lexicon of cultural protective factors and to adapt a Community Needs Assessment (CNA). *Phase 2* enrolls 90 Shawnee adults in a simultaneous functional MRI/electro-encephalography (fMRI/EEG) battery comprising culturally tailored picture and audio paradigms, a Monetary Incentive Delay (MID) task, a three-arm bandit task, and the Horizon exploration task. Self-report scales assess cultural identity, mental health, substance use, impulsivity, and risk. Parallel CRA interviews (*n* = 12) quantify community readiness for SUD interventions across six dimensions. *Phase 3* will recruit Shawnee citizens with lived SUD experience to examine whether cultural, behavioral, and neural markers predict recovery-related outcomes (e.g., craving, self-efficacy).

**Discussion:**

By integrating community insight with state-of-the-art neuroimaging under Tribal governance, this protocol addresses critical knowledge gaps in Indigenous SUD research, models CARE-aligned data stewardship and establishes a transferable framework for culturally grounded precision substance use prevention.

## 1 Introduction

Substance-use disorders (SUDs) remain a leading cause of preventable morbidity and mortality in the United States. Recent CDC surveillance indicates the age-adjusted drug-overdose mortality rate among American Indian and Alaska Native (AI/AN) peoples reached 65.2 deaths per 100 000 in 2022—double the general U.S. rate of 32.6 and the largest year-over-year increase of any racial/ethnic group (1) and in the 2023 National Survey on Drug Use and Health, 25.3% of AI/AN adults met criteria for a past-year SUD, a disproportionately high rate of suffering (2). Oklahoma, where the Shawnee Tribe is headquartered, reported a 2022 overdose-mortality rate of 30.7 per 100,000, exceeding the national average (3). The root causes of substance-use disparities likely come from longstanding environment and contextual factors such as disenfranchisement, assimilation policies, geographical removal, and erosion of traditional lifeways (4). These factors complicate engagement with mainstream treatment services and modern approaches to substance use research efforts such as psychiatric neuroscience. In the current protocol, cultural factors are conceptualized as core exposome variables (e.g., language use, community connectedness, ceremonial participation, kinship, spirituality) that interact with behavioral and neural systems to foster resilience across the life course. This conceptualization enables the field to move beyond broad and non-specific definitions of “culture” as a protective factor, by providing operational definitions of specific cultural factors. Furthermore, Tribal perspectives underscore the restorative value of ceremony, language reclamation, and community events for recovery and mental-health promotion. In turn, addressing substance-use morbidity and mortality necessitates community-informed and culturally appropriate research efforts that combine deep engagement with state-of-the-art neuroscience.

Recent pilot work from our group illustrates the promise of this integrative approach. Activation of inhibitory control networks (i.e., dorsolateral prefrontal cortex) was found among individuals with no reported risk for suicide relative to those with some reported suicidal thoughts and behaviors (5). Additionally, activation in the IFG during inhibitory control tasks differentiated individuals with and without SUD diagnoses (5). Our work has also demonstrated that individuals with lifetime substance use display reduced sensitivity to high magnitude loss indicated by lower blood oxygenation level dependent signal response in the striatum relative to healthy individuals (6). Furthermore, we have shown that self-reported Native American spirituality modulated cognitive control disruption (indexed by P3 amplitudes the stop-signal task) in individuals with generalized anxiety disorder (7). A follow-up study extended these findings to demonstrate that Native American spirituality and social support are associated with increased cognitive efficiency in AI adults during a response inhibition task (8). These findings indicate that culturally rooted protective factors are related to measurable signatures in brain systems implicated in SUD risk underscoring the rationale for a community-engaged, multimodal design that can quantify specific neural signatures across levels of analysis and provide a more precise operationalization of culturally relevant salutogenic factors.

Community-engaged multi-method research, including multimodal neuroimaging and community grounded qualitative inquiry, weaves together community narratives, behavioral evidence, and brain-based biomarkers into a single explanatory fabric. This novel integrative strategy is uniquely suited to substance-use research with AI/AN populations, where culturally grounded protective factors are often unmeasured and or ignored in standard clinical measurement tools, yet fundamental to resilience. The current project can illuminate modifiable pathways by first defining these factors through qualitative inquiry and subsequently operationalizing them using concurrent fMRI/EEG paradigms. Deep engagement with tribal partners and governing infrastructure ensures findings remain tribally relevant and compatible with tribal sovereignty and autonomy. Beyond regulatory and ethical considerations, the CBPR approach to empirical multimodal neuroimaging research provides pathway to clinical impact which are novel to the field by tethering the investigation to community goals and priorities enhancing the likelihood of meaningful and actionable outcomes.

**Figure 1:**
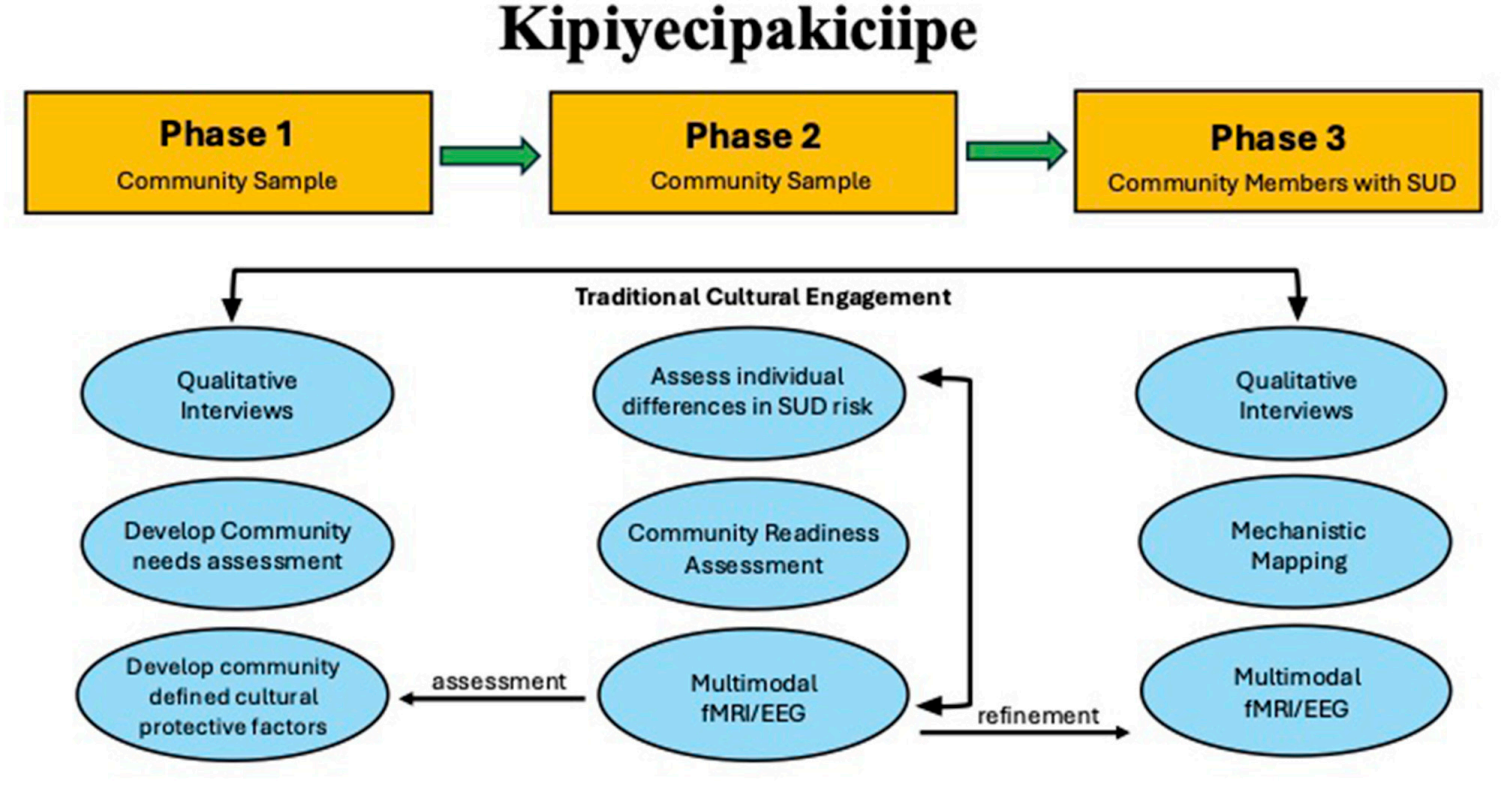
This figure shows the overall phases of the protocol and components of each.

*Kipiyecipakiciipe* “coming home” embodies this logic as a three-phase multi-method CBPR research protocol that incrementally links community knowledge, advanced neuroimaging, and actionable service partnerships (Figure 1). Phase 1 focuses on community-defined cultural protective factors via qualitative research methods. Trained facilitators conducted a series of focus group discussions (FGD; including nominal group technique; 5–10 citizens per group) to elicit narratives of how traditional cultural engagement (TCE) fosters wellness and substance-use resilience. Nominal group technique was utilized in one FGD to generate, share, discuss, and prioritize a list of Shawnee traditional ways (STW) that support wellbeing. Reflexive thematic analysis of discussions and the prioritized list of Shawnee traditional ways that support wellbeing generated an operational lexicon of cultural protective factors that now anchors the quantitative measures and informed stimulus development for neuroimaging paradigms employed in later phases. Phase 2 comprises three parallel protocols: (i) the primary protocol is a multimodal fMRI/EEG protocol with 90 adults to test neural signatures of the Phase 1 factors as well as self-reported levels of measurement for the operationalized constructs; (ii) a Community Readiness Assessment (CRA): an adapted needs assessment using the Community Readiness Model (9) providing immediate benefit to Shawnee Tribe capacity by providing an assessment related to current resources, perspectives, and needs for SUD prevention and intervention programing in the community. This process also formalizes recruitment pipelines for clinical sampling that may be used in Phase 3; and (iii) an extension of Phase 1 Qualitative work to individual qualitative interviews with citizens who have lived SUD experience to refine the operationalized factors of cultural engagement for relevance to recovery contexts. In Phase 3, insights from Phase 2 neuroimaging, CNA partnerships, and qualitative interviews converge to inform the design of a second multi-modal neuroimaging study focused on citizens in with lived experience with SUD, enabling mechanistic mapping of how TCE supports sustained recovery and resilience. Furthermore, empirical data from phase 2 regarding multilevel SUD risk factors will refine the self-report, neural, and behavioral clinical targets of the phase 3 protocol.

Although a growing body of scholarship advocates community-engaged, strengths-based, and culturally grounded mental-health research with American Indian (AI) populations, the existing empirical base remains predominantly descriptive. A 2024 mixed-studies review of 343 records identified only 19 quantitative papers measuring risk or protective factors among AI youth and none included neurobiological outcomes (10). A complementary scoping review of culture-based addiction interventions likewise found that most evaluations relied on small, quasi-experimental designs with heterogeneous outcome measures, limiting mechanistic insight (11). To date, no published work has combined phased multi-method inquiry with multimodal neuroimaging in direct partnership with a sovereign Tribal Nation through CBPR. The few neuroimaging papers that do include AI/AN participants focus almost exclusively on age-related cerebrovascular disease or dementia and are conducted in non-sovereign clinical settings (12, 13). Consequently, the field still lacks evidence on how community-defined cultural exposures map onto neurobehavioral processes relevant to substance-use-disorder (SUD) risk and resilience. Beyond the scientific gaps, AI/AN leaders emphasize Tribal data sovereignty is necessary to prevent extractive research and ensure reciprocal benefit. A 2024 scoping review of routinely collected health-data studies found that only 21% referenced Indigenous guiding principles and just 41% mentioned data sovereignty, underscoring limited compliance with the CARE framework (14). Recent commentaries underscore the importance of designing and conducting studies that honor tribal sovereignty (15).

The current protocol is co-developed with and overseen by partners from the Shawnee Tribe. Together, these contributions position Tribal sovereignty at the center of cutting-edge neuroscience and establish a scalable framework (i.e., community definition, neurocognitive testing, clinical sample validation) that can generalize to other communities and accelerate community-driven discovery for solutions to SUD. The overarching goal of *Kipiyecipakiciipe* is to establish Shawnee-defined cultural engagement factors and how they confer resilience against substance-use disorder. To accomplish this, the project follows three mutually informative phases. In sum, the study advances three linked hypotheses: (i) Shawnee community members articulate discrete, operationally definable cultural factors that can be quantified; (ii) these factors are associated with neurobehavioral indices implicated in self-regulation, decision making, and salience processing; and (iii) the resulting brain–behavior signatures forecast resilience and recovery trajectories, thereby informing culturally aligned prevention and treatment strategies.

## 2 Methods

### 2.1 Study overview

Across all phases, the study integrates culturally grounded qualitative, CBPR, and neuroscientific methods to explore how TCE supports mental health and substance recovery among Shawnee citizens. Findings will inform the development of sustainable, community-led prevention and intervention strategies rooted in Shawnee cultural strengths.

*In Phase 1* we have already collected and analyzed qualitative data to understand cultural activities and engagement that support Shawnee wellbeing and substance use resilience. The qualitative analysis produced an operational lexicon of cultural protective factors and yielded an adapted Community Needs Assessment (CNA) instrument tailored to local clinical services.

*In Phase 2* we translate these community-defined factors into quantifiable factors through a multimodal neuroimaging protocol, we will test whether scores on identified cultural scales moderate associations between SUD relevant individual difference factors (e.g., impulsivity, delay discounting) and neurocognitive markers of reward and loss processing and decision making as well as computational parameters of behavioral decision-making tasks. Additionally, community defined factors are used to develop cultural stimuli protocols to map neural circuits and markers of cultural identity processing. In addition to moderating effects, we anticipate cultural self-report scales are associated with activation of self-referential processing networks. Parallel deployment of a community readiness assessment in the ST community and behavioral health settings will map readiness profiles and formalize recruitment pipelines for the next phase. Additionally, a second wave of qualitative data focused on individual interviews with citizens with lived experience with SUD will refine the target cultural factors from Phase 1 to those most relevant for SUD recovery in Phase 3.

*Phase 3* will integrate what we learn to examine whether the combination of cultural, behavioral, and neural markers predicts key recovery outcomes among citizens with lived SUD experience. We anticipate that level of cultural factors will mediate the association between behavioral risk related constructs (e.g., impulsivity and delay discounting) as well as neural activation patterns and outcomes such as craving intensity and self-efficacy, providing specific and culturally relevant intervention targets.

### 2.2 Setting and tribal governance

This work is conducted in collaboration with the Shawnee Tribe, a sovereign Tribal Nation headquartered in Oklahoma, with most citizens residing in northeastern Oklahoma. The study operates under governance procedures of the Tribe, with oversight provided by the Shawnee Tribe Business Council and a Community Advisory Board (CAB) composed of cultural knowledge bearers. The CAB, which includes nine Shawnee adults, was formed in partnership with Tribal leadership and provides ongoing oversight, co-development of study materials, and review of all findings to ensure cultural and contextual accuracy. All procedures align with CBPR principles and ensure Shawnee Tribe authority and autonomy with respect to cultural data.

Study activities are conducted in private offices either at LIBR in Tulsa, Oklahoma, or in community facilities with controlled access to ensure participant confidentiality. Oversight includes approval from the WCG Institutional Review Board (IRB) and formal agreements with the Shawnee Tribe Business Council approval. The Tribe retains authority over data collected from Shawnee citizens, including any future use or archiving. All publications, presentations, and reports are submitted for CAB review prior to external dissemination. The research team also holds quarterly consultation meetings with the CAB to maintain transparency, adapt study procedures as needed, and uphold principles of Tribal sovereignty throughout the project.

### 2.3 Participants and recruitment

Participant groups are recruited for each study phase using sampling methods appropriate to each protocol and with respect to cultural protocol where necessary. Monetary compensation is provided across all phases via reloadable ClinCard. Compensation level and communication thereof are consistent with prior studies and reasonable to ensure that participant incentives do not bias recruitment. Compensation plan was reviewed and approved by the IRB and CAB. Importantly, participation in one study protocol is orthogonal to other protocol, it does not necessitate or preclude participation in other phases/protocols. Participants provide written informed consent prior to participation in each of the study protocols.

#### 2.3.1 Phase 1 (qualitative exploration of traditional cultural engagement)

Twenty two culturally engaged adult Shawnee citizens were recruited using purposive sampling in collaboration with Tribal staff through community networks, and word-of-mouth. Specifically, the CAB indicated specific individuals you recruit based on the community level understanding of the potential participants as knowledgeable about traditional cultural practices. Participant inclusion criteria were 18 years or older, self-identify as culturally engaged, and be willing to participate in group or individual interviews. No individuals identified through purposive sampling were excluded for not meeting inclusion criteria or any other reasons. The study team and CAB discussed holding separate focus groups between these two genders because STW may differ by gender and role. This was done to ensure that participants would feel safe and comfortable sharing, and to prevent any discomfort related to cultural protocols around discussing or sharing one gender's role around another. FGDs were audio-recorded and transcribed for analysis. Following completion of the FGDs, the research team discussed and wrote memos.

#### 2.3.2 Phase 2a (**community readiness assessment)**

Twelve community stakeholders were selected via purposive sampling to reflect broad community perspectives consistent with recommendations from the Community Readiness Model (9) including young adults (ages 18–25), elders, service providers, law enforcement officials, and Tribal leaders. Individuals were nominated by Shawnee Tribe staff or CAB members. Specifically, ST behavioral health staff, CAB members, and LIBR staff submitted recommendations for review by the CAB, which made final determinations about inclusion eligibility. The CAB consultation ensured appropriateness in participant selection and data interpretation. Each participant was at least 18 years old and consented to a recorded interview. All interviews will be conducted by trained Shawnee Tribe Behavioral health department staff or LIBR study staff guided by standardized protocols.

#### 2.3.3 Phase 2B (Shawnee tribe scanning study)

Ninety participants (aged 18 and older; any gender) representative of ST adult population will be recruited from the Shawnee community. A sample size of 90 participants provides adequate power (80% at α = 0.05) to detect moderate to small effect sizes (*d* = 0.27–0.33) in within subject linear mixed-effect models examining interaction of a risk and resilience variable on a neurobehavioral and or symptom outcome. Furthermore, this sample with enable effect size estimation for smaller effects important to explore in future research the sample is not powered to estimate robustly. This is important considering many clinically important variables demonstrate small effect sizes (16). For eligibility participants must have been an enrolled Shawnee citizen and/or active in the traditional Shawnee community. Recruitment methods included in-person outreach, web-based sign-ups (e.g., social-media), tribal newsletter announcements, and phone screening coordinated by trained research staff. Exclusion criteria included severe or unstable psychiatric or medical conditions, cognitive or developmental disorders, and contraindications for MRI. Participants completed MRI, EEG, behavioral tasks, and surveys in a single 3.5 h session. All procedures followed safety and data privacy protocols from the LIBR IRB scanning protocol.

#### 2.3.4 Phase 3 (assessment of traditional cultural engagement in individuals with SUD history)

Participants will include Shawnee citizens—those with substance use disorders (SUD) history and healthy controls (HC), recruited in partnership with Shawnee Tribe treatment services, legal systems, and broader community networks. Whereas, phase 2 is a general sample exploration of potential risk and resilience pathways, Phase 3 aims to elicit specific clinically relevant insights in risk and resilience pathways for individuals with SUD. Procedures will mirror those in Phase 2, with flexibility to adapt based on ongoing community input and participant needs. Furthermore, the protocol will focus on behavioral and neuroimaging tasks with effects that demonstrate most promising risk and resilience effects from Phase 2 to reduce participant burden in the clinical sample.

### 2.4 Procedures and analyses

#### 2.4.1 Phase 1

The first qualitative component explored how cultural knowledge and participation are conceptualized by Shawnee citizens and how these experiences relate to health, healing, and substance use resilience. Three focus group discussions (FGDs) were conducted using purposive sampling. One group was composed of the CAB and subsequently two gender-stratified groups were used to respect cultural protocols and ensure participant comfort.

Facilitated by the project PI and co-investigators along with two notetakers and support staff, the CAB FGD included nominal group technique (NGT) (17) to identify and prioritize Shawnee traditional ways (STW) that promote wellbeing. Each focus group was guided by community-developed questions about engagement with traditional Shawnee culture and its role in promoting wellbeing and mitigating substance misuse. Following FGDs, one key informant in-depth interview was conducted in order to include a key informant (i.e., tribal elder) who was not able to be present for the FGD. The scope and structure of FGDs and IDI were determined collaboratively with the CAB to ensure cultural relevance and appropriateness.

FGDs and the IDI were audio-recorded with participant consent, transcribed (removing identifiers), and the recordings were destroyed following transcription.

The analysis plan includes a brief summary of the project, methods, and data collection. The plan articulates the goals of the analysis as: (1) Produce a list of Shawnee traditional ways (STW) with contextual information to inform development of behavioral and neural probes for use in phase 2 of the larger research project; (2) Answer the research questions: (a) how do STW impact and relate to wellbeing for Shawnee people and their relatives; and (b) How do STW impact and relate to substance use among Shawnee people and their relatives; and (3) Conduct an exploratory analysis to honor all of the community members who shared their valued perspectives with us. To develop a codebook, authors FG, MC, VMO, and EJW reviewed the FGD with CAB members to produce some initial code ideas. FG and MC used these discussions to create a version 1 codebook which was shared with the team for review. Next, coders FG and MC tested codebook version 1 using the transcript from one FGD. FG and MC independently began applying the codebook to these transcripts using Atlas.ti, a software used to manage qualitative data analysis. Throughout this process the codebooks is finalized and applied to all transcript.

#### 2.4.2 Phase 1 analysis

NGT data was analyzed in real-time during FGD breaks, allowing the study team to then display the list of Shawnee traditional ways and facilitate discussion. Two additional gender-specific FGDs did not engage in an NGT activity, but were provided with the initial list to discuss and generate additional Shawnee traditional ways to be included with respect for safety and comfort in adding gender-specific traditional ways. The final generated list of Shawnee traditional ways from all three FGDs was reviewed and approved by the CAB. De-identified transcripts were then used for reflexive thematic analysis, with coding developed inductively and interpreted in partnership with CAB members. The final list of Shawnee traditional ways and themes informed both construct development and the future design of behavioral and neuroimaging tasks and stimuli in subsequent study phases.

#### 2.4.3 Phase 2a community readiness assessment (CRA)

The CRA assesses the Shawnee Tribe's readiness to implement and support substance use prevention and intervention efforts. Grounded in the CRM9 the CRA evaluates six key dimensions on a nine-stage continuum (Table 1).

**Table 1:** Community readiness assessment (dimensions and stages).

**Dimensions of community readiness**
1. Community efforts: the extent to which programs, activities, and policies exist that address the issue (i.e., substance use prevention or mental wellness). This includes both formal and informal efforts
2. Community knowledge of the efforts: the level of awareness among community members regarding existing efforts, including their effectiveness and accessibility to all community segments
3. Leadership: the degree of support and involvement from appointed or elected official and informal leaders (i.e., elders, cultural leaders) regarding the issue
4. Community climate: the prevailing community attitudes, norms, and receptiveness toward addressing the issue. This reflects the general openness to discussing and engaging with the problem
5. Community knowledge about the issue: the extent to which the community understands the issue's causes, consequences, prevalence, and implications, and the degree to which accurate local data are available and disseminated
6. Resource and prevention: the availability of people, time, money, space, and other resources within the community to support prevention or intervention efforts. This also includes community willingness to mobilize those resources
**Stages of community readiness**
1. No awareness: the issue is not generally recognized by the community or leaders as a problem
2. Denial/Resistance: some recognition that the issue exists, but little recognition that it occurs locally or affects the community
3. Vague awareness: there is acknowledgment of a local problem, but no motivation to act. Understanding is stereotyped or imprecise
4. Preplanning: the issue is clearly recognized as a problem. Discussion has begun, but no detailed planning has occurred yet
5. Preparation: planning efforts are underway. Specific activities or policies are being considered. Community support is modest
6. Initiation: activities have been launched and are gaining traction. They are viewed as new efforts with limited evaluation
7. Stabilization: programs or efforts are established and stable, Staff are trained, and evaluation is beginning. There is institutional support
8. Confirmation/expansion: programs are evaluated and improved. Efforts expand, and community awareness and support are high. New needs are being addressed
9. High level of community ownership: the community demonstrates comprehensive knowledge of the issue. Programs are well-integrated, routinely evaluated, and community-led

Twelve semi-structured interviews will be conducted with key representatives across community sectors using an interview guide adapted from the CNA Interview Questions document.

Interview questions spanned all six readiness dimensions and were adapted to reflect substance use concerns in the Shawnee community. Each interview is anticipated to last approximately 30–60 min and will be audio recorded, transcribed, and de-identified prior to scoring. Interviewers were trained to avoid leading responses and to prioritize community voice and perception throughout. Scoring will be carried out by two raters with formal training in the CRM. Each reviewer independently read the full set of interview transcripts and applied the anchored rating scales to assign scores across the six dimensions. After initial scoring, the raters met to compare assessments and reach consensus on each dimension, producing final readiness scores for the community.

The CRA process will both guide research protocol development with respect to clinical sampling in Phase 3 and provided immediate community benefit in the form of a needs assessment report. Specifically, the protocol will inform clinical services by identifying readiness barriers and opportunities, strengthen relationships with local clinical and service providers, and supported recruitment infrastructure for Phase 3 of the study. Additionally, findings will guide culturally appropriate program development and support iterative adaption of future community interventions.

#### 2.4.4 Neuroimaging empirical protocols

After providing informed consent participants (target *n* = 90) will complete a battery of questionnaires assessing a range of individual difference factors including cultural factors, risk and resilience factors, and substance use symptom measures. Participants will also complete a behavioral decision-making task. Finally, participants will complete cultural stimulus tasks (i.e., picture viewing and audio), monetary incentive delay (MID) task (18), and three arm bandit task during concurrent EEG/fMRI recording.

The Horizon task, used in previous computational work (19), evaluates directed vs. random exploration in a two-armed setting. Participants play 80 games, evenly divided between Horizon 1 (one free choice after four forced samples) and Horizon 6 (six free choices after the same four forced samples). Option pay-outs are Gaussian (1–100 points, SD = 8) with mean differences of 4–30 points that remain constant within a game. By comparing choice behavior in H6 vs. H1 trials, the task yields indices of strategic information seeking under different time horizons; bonuses are paid in addition to participant compensation.

The MID (18) comprises 90 trials presented during fMRI. On each trial a visual cue indicates whether the participant can gain money, avoid losing money, or experience no monetary change. After a brief interval, a target appears and the participant must respond before an individually titrated deadline that yields ≈ 66% success. Outcome feedback immediately follows. Cues are counter-balanced across valence (gain, loss, and neutral) and three relative magnitudes—low, moderate, and high.

In the three-arm bandit task, modeled on previous empirical work (20), participants complete 20 independent games, each containing 16 trials. At the start of every game, the three options are assigned stable-but hidden—reward probabilities that were pre-generated from a Beta (2, 2) distribution and remain fixed for that game only. On each trial the participant selects an option (≈ 2 s limit) and receives feedback 250 ms later: a green token (1 point) or red token (0 points). Reward probabilities reset between games, encouraging early exploration followed by exploitation. Performance-based bonuses of paid according to cumulative points.

The CAB co-developed culturally specific imaging picture and audio stimuli similar to our previous work (21). For the cultural picture task, 110 candidate images depicting Shawnee cultural themes were rated (0–100) for cultural connectedness; the 48 highest-scoring images were retained and matched to 48 comparator images on low-level visual properties. The cultural audio task comprises a single ≈4-min recording of traditional Shawnee music approved by the CAB.

#### 2.4.5 MRI and simultaneous EEG acquisition

Scanning is performed on a 3 T Siemens Prisma scanner: gradient-echo EPI (TR = 2,000 ms, TE = 27 ms, flip = 70°, 40 axial slices, 1.875 × 1.875 × 2.9 mm voxels) and high-resolution T1-weighted MPRAGE (0.9 mm isotropic). Pre-processing in AFNI includes despiking, slice-timing and motion correction, Talairach normalization, and 6 mm FWHM smoothing. First-level GLMs model task regressors with a 4–6 s peak HRF; motion and low-frequency trends are nuisance covariates. Percent signal-change values from *a priori* ROIs feed group-level analyses.

Simultaneous 32-channel EEG (BrainAmp MR Plus; 5 kHz, 0.016–250 Hz) uses a 10–20 layout (reference FCz, ground AFz). EEG assists retrospective head-motion correction (E-REMCOR) and ERP extraction (e.g., N200). Data will be cleaned with standard processing pipeline (22), band-pass filtered 0.01–35 Hz, down-sampled to 250 Hz, re-referenced to average-mastoids, epoched −200–800 ms, and automatically artifact-rejected; datasets with >25% rejected trials are excluded.

## 3 Dissemination plan

Findings will be disseminated first to Shawnee CAB members through quarterly briefings and dissemination plans will then be co-developed between CAB and LIBR study team. These plans will then be submitted for review and approval by the ST Business Council. Academic dissemination will follow, ensuring Shawnee Tribe authorities approve all manuscripts conference materials and prioritizing open-access venues to ensure published resources are accessible directly to the community. Lay summaries and infographics will be provided to the Tribe and archived according to ST priorities and discretion.

## 4 Discussion

The current project offers the first sovereignty-aligned neurobehavioral protocol designed to examine of how to operationally define facets of traditional cultural engagement and test modulations of salience processing, decision making, and self-regulatory processes implicated in SUD risk. By integrating community defined factors of cultural engagement into a rigorously designed multimodal neuroimaging (fMRI/EEG) protocol the study aims to translates community defined cultural knowledge from narrative insight to neurobehavioral mechanism, as a first step in culturally responsive precision medicine.

In addition to scientific advancement, the project delivers concrete, near-term benefits to community partners through thoughtful design. Employing the Community Readiness Assessment provides a tangible needs assessment for behavioral health program development in the partner community, while the project's cultural factor operationalization provides a foundation for developing a culturally grounded prevention programming. This ensures that the process is beneficial to the Tribal partners by design not only potential findings in the empirical work which are unlikely to yield immediate and direct public health benefit due to the nature of clinical neuroimaging research. All primary data remain Shawnee-owned; any secondary use requires CAB and business council approval, providing a CARE-compliant study protocol model that other community engaged research groups can adopt.

A key innovation lies in the reciprocal flow between qualitative insight and neuroimaging protocol development. Prior Indigenous-health scholarship has typically separated community perspectives from laboratory science, resulting in measurement tools with limited functional specificity and neurobiological studies that seldom incorporate community-defined priorities (10, 11, 23). We counter this trend by weaving Shawnee-defined protective factors through every step of the project, from constructing operational definitions and culturally grounded stimuli to interpreting imaging outcomes. In parallel, the CNA delivers immediate, practical benefit to Shawnee behavioral-health services, illustrating a model of *regenerative* rather than *extractive* science (14, 23). Methodologically, our single-cohort integration of functional MRI, structural MRI, EEG, and computational modeling offers a multilevel lens rarely achieved in AI/AN research and sovereignty-aligned governance keeps all findings under Tribal control.

### 4.3 Limitations

Several limitations warrant consideration. First, although the Phase 2 cohort (*N* = 90) exceeds sample sizes of most existing Indigenous neuroimaging studies to our knowledge, statistical power to detect small effect sizes or complex interactions may be limited. We mitigate this by focusing hypotheses on medium to large effects derived from prior pilot data (5, 7, 8, 24). Furthermore, employing Bayesian estimation to quantify evidence for null vs. alternative models can also reduce the impact of this limitation. Second, TCE measurement rooted in extensive qualitative groundwork, remains susceptible to limited direct generalizability beyond the Shawnee context. However, the phased framework is intentionally modular and designed in such a way that other study groups and populations can substitute their own contextual factors and governance structures. This is generalizable framework for expanding (1) the scope of constructs assessed in typical neuroimaging protocols and (2) quantification of biological indicators of functional processes that have been largely inaccessible to the community-engaged health literature.

### 4.4 Future directions

Future directions include (1) extending the exposome-neurocognitive model to adolescent cohorts to capture developmental windows of opportunity; (2) leveraging flexible measurement modalities (e.g., mobile EEG and ecological momentary assessment, passive data collection) to map dynamics of identified constructs and effects in ecologically valid contexts; (3) evaluating resultant neurobehavioral markers for predictive value in culturally adapted clinical programs; and (4) collaborating with other AI/AN populations to test the portability of the developed framework. Ultimately, the study aspires to demonstrate that community-engaged neuroscience can develop actionable pathways honoring Tribal sovereignty and knowledge systems to address SUD disparities while also expanding the reach of neuroscience to constructs and dynamics previously unaccounted for in the literature.
